# 

**Published:** 1876-03

**Authors:** 


### Table of Contents.

### ORIGINAL COMMUNICATIONS.

Urethritis—Its Treatment. *By Jno. Thad. Johnson, M.D.* .......... 705
Ovulation and Menstruation, and Dr. Battey's Operation of Normal Ovariotomy. *By Ch. Rauschenberg, M.D.* .......... 713
Fluor Albus. *By J. G. Westmoreland, M.D.* .......... 728
Influence of Climate on Pectoral Diseases. *By J. G. Westmoreland, M.D.* .......... 729

### ATLANTA ACADEMY OF MEDICINE.

Opium Poisoning, 731—Melanotic Cancer, 735—Belladonna in Contraction of Sphincter, 735—Poisoning from Mouse Meat, 736—Worms, 737—Pruritus Ani .......... 738

### CLINIC ATLANTA MEDICAL COLLEGE.

Services of A. W. Calhoun, M.D .......... 740
Presbyopia, 740—Acute Catarrh of the Middle Ear, 741—Double Strabismus, 742—Obstruction of Middle Ear, 742—Deafness of Fifteen Years .......... 743

### OUR EXCHANGES.

Communicability of Diphtheria .......... 744
Removal of Cherry-Stones from the Rectum .......... 746
Salicylic Acid in Rheumatism .......... 747
Ergot in Purpura Hæmorrhagia .......... 748
Labor Operation .......... 749
Anæsthetic in New Form .......... 750
When to Bleed or not .......... 752
How to Prevent Chloroform Asphyxia .......... 755
Styptic Cotton in Otorrhœa .......... 756
Gelsiminum in Cough .......... 757
Salacine in Diarrhœa .......... 757

### BIBLIOGRAPHICAL.

Polar and Tropical Worlds .......... 758
Lectures on Diseases Nervous System .......... 757
Cyclopædia of Practice of Medicine .......... 758
Pamphlets .......... 759

### EDITORIAL.

State Board of Health .......... 760
Atlanta Medical College .......... 761
Abstract .......... 762

